# AAV capsid engineering identified two novel variants with improved *in vivo* tropism for cardiomyocytes

**DOI:** 10.1016/j.ymthe.2022.07.003

**Published:** 2022-07-09

**Authors:** Laura Rode, Christian Bär, Sonja Groß, Axel Rossi, Nadja Meumann, Janika Viereck, Naisam Abbas, Ke Xiao, Isabelle Riedel, Anika Gietz, Karina Zimmer, Margarete Odenthal, Hildegard Büning, Thomas Thum

**Affiliations:** 1 Institute of Molecular and Translational Therapeutic Strategies (IMTTS), Hannover Medical School, OE 8886, Carl-Neuberg-Str. 1, 30635 Hannover, Germany; 2 REBIRTH Center for Translational Regenerative Medicine, Hannover Medical School, 30625 Hannover, Germany; 3 Institute of Experimental Hematology, Hannover Medical School, 30625 Hannover, Germany; 4 Institute of Pathology, University Hospital of Cologne and Center for Molecular Medicine Cologne, University of Cologne, 50937 Cologne, Germany; 5 Fraunhofer Institute for Toxicology and Experimental Medicine, 30625 Hannover, Germany

**Keywords:** gene therapy, vector engineering, adeno-associated virus, heart, cardiac hypertrophy, non-coding RNA, AAV library screening, AAV vectors, AAV2, lncRNA H19

## Abstract

AAV vectors are promising delivery tools for human gene therapy. However, broad tissue tropism and pre-existing immunity against natural serotypes limit their clinical use. We identified two AAV capsid variants, AAV2-THGTPAD and AAV2-NLPGSGD, by *in vivo* AAV2 peptide display library screening in a murine model of pressure overload-induced cardiac hypertrophy. Both variants showed significantly improved efficacy in *in vivo* cardiomyocyte transduction compared with the parental serotype AAV2 as indicated by a higher number of AAV vector episomes in the nucleus and significant improved transduction efficiency. Both variants also outcompeted the reference serotype AAV9 regarding cardiomyocyte tropism, reaching comparable cardiac transduction efficiencies accompanied with liver de-targeting and decreased transduction efficiency of non-cardiac cells. Capsid modification influenced immunogenicity as sera of mice treated with AAV2-THGTPAD and AAV2-NLPGSGD demonstrated a poor neutralization capacity for the parental serotype and the novel variants. In a therapeutic setting, using the long non-coding RNA *H19* in low vector dose conditions, novel AAV variants mediated superior anti-hypertrophic effects and revealed a further improved target-to-noise ratio, i.e., cardiomyocyte tropism. In conclusion, AAV2-THGTPAD and AAV2-NLPGSGD are promising novel tools for cardiac-directed gene therapy outperforming AAV9 regarding the specificity and therapeutic efficiency of *in vivo* cardiomyocyte transduction.

## Graphical abstract

**Figure:**
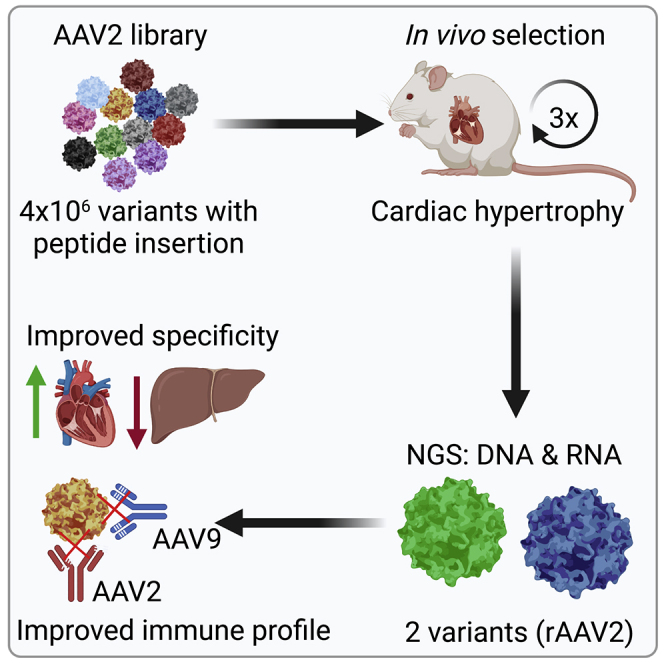

## Teaser

Gene therapeutic approaches for heart diseases are limited by the unavailability of efficacious delivery tools. Rode et al. report on AAV2-based cardiotropic capsid variants. Through improved cardiomyocyte transduction and liver de-targeting, anti-hypertrophic effects of lncRNA *H19* were obtained with more than a log lower vector doses compared with conventional approaches.

## Introduction

Adeno-associated virus (AAV) vectors are frequently used delivery tools for experimental, preclinical, and clinical *in vivo* gene transfer.^1^ Hitherto, three AAV vector-based gene therapies have received market authorization in Europe and more are expected soon based on promising data from various clinical trials.^1^^,^^2^

The parental virus delivers a single-stranded DNA genome of approximately 5 kb, which is flanked at either end by inverted terminal repeats (ITRs). The *rep* gene, located in the 5′ part of the genome, encodes for a family of multifunctional non-structural proteins involved in the packaging, replication, and integration of the wild-type virus genome.^3^ The *cap* gene delivers—in addition to the assembly activating protein and the membrane-associated accessory protein—coding information for three structural proteins: the major capsid viral protein (VP) VP3, as well as the two *N*-terminally extended minor forms VP1and VP2. The common VP3 regions of the three capsid proteins form the actual capsid with characteristic structures such as protrusions around the three-fold and channel-like pores at the five-fold axis of symmetry.^4^^,^^5^ Naturally occurring AAV serotypes that differ in capsid amino acid sequences, impacting tropism and immunogenicity, are in use as first-generation vectors in human gene therapy.^6^

In the cardiovascular field, AAV serotype 1 (AAV1) vector-based clinical trials, in particular the phase I and II CUPID trials (calcium upregulation by percutaneous administration of gene therapy in patients with cardiac disease) demonstrated safety, but showed no clinical efficacy.^7^^,^^8^ The latter was most likely caused by insufficient transduction of cardiomyocytes, since less than 2% of cardiomyocytes contained a vector genome and thus would have been able to express the transgene.^1^^,^^9^ Alternative serotypes were explored in rodents as well as in large animal models, such as sheep, pigs, and non-human primates.^10^^,^^11^^,^^12^ The efficacy differed depending on the administration route and the animal model. In rodents, for example, a 20-fold and 200-fold higher level of cardiomyocyte transduction compared with AAV1 was reported for intravenously applied AAV8 and AAV9, respectively, and AAV9 is also the preferred serotype for skeletal muscle transduction in mice.^13^ In contrast, AAV6 as well as AAV1 were superior when applied intramyocardially or intrapericardially.^13^ In large animal models, AAV6 and AAV9 showed promise, with AAV6 being superior after a trans-endocardial or intra-coronary administration and AAV9 after intravenous administration.^14^ However, AAV9 like other serotypes accumulate in the liver after intravenous injection.^15^^,^^16^ To compensate for off-target transduction, high vector doses are applied to sufficiently target the heart, which in turn increases the risk of undesired and potentially fatal side effects.^17^ Thus, there is an urgent need for AAV vectors with improved cardiac tropism.

By replacing a hexapeptide containing the heparan sulfate proteoglycan (HSPG) binding motif of AAV2 located at the tip of the second highest protrusion with the corresponding residues of AAV8, a capsid variant—termed AAV2i8—was developed which met these features, i.e., improved whole-body muscle as well as cardiac tropism combined with liver de-targeting.^18^ This variant is currently being tested in a first-in-man human clinical trial for heart failure with reports on improvements in the left ventricular ejection fraction and New York Heart Association functional classification (abstract ASGCT 2022^19^).

Here, we focused on the same serotype, but followed a different approach. Instead of replacing the naturally occurring hexapeptide of AAV2 with AAV8, we screened *in vivo* in mice a library of AAV2 capsid variants displaying at the top of the same protrusion a heptamer insertion of random sequence. We thereby identified AAV2-THGTPAD and AAV2-NLPGSGD, two capsid variants with improved tropism for cardiomyocytes accompanied with improved intra-cellular processing, all compared with the parental AAV2 serotype. *In vivo* cardiomyocyte transduction of our novel variants is reaching levels comparable with AAV9, while outperforming this serotype concerning liver de-targeting.

## Results

### *In vivo* selection of an AAV2 peptide display library on hypertrophic cardiomyocytes resulted in enrichment of specific variants

**Figure 1:**
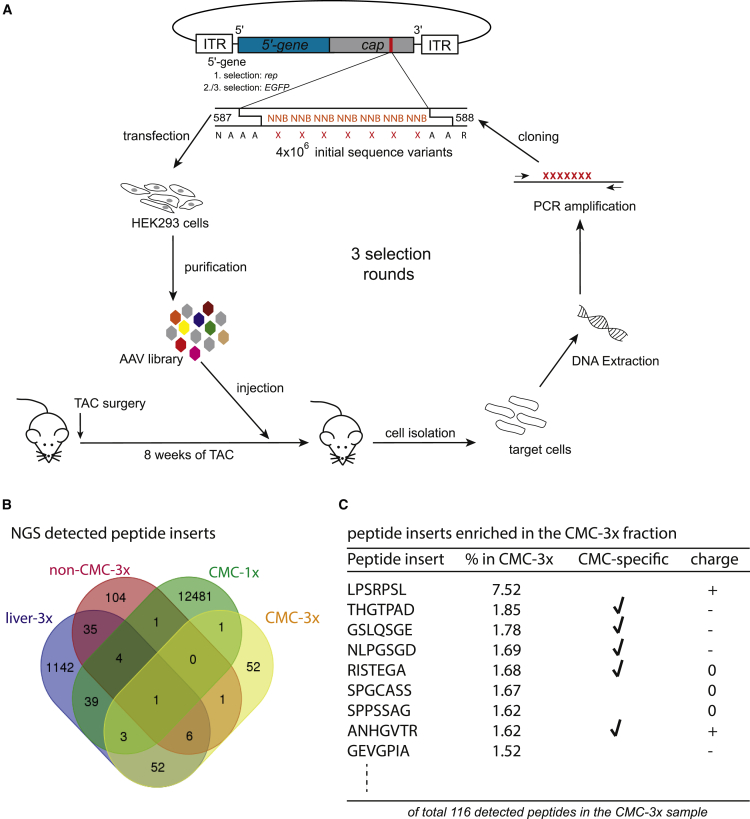
AAV peptide display library screening on hypertrophic cardiomyocytes *in vivo* (A) Schematic illustration of the library screening. (B) NGS results illustrating the peptide inserts detectable after three rounds of selection in the CMC fraction (CMC—3×), in the non-cardiomyocyte fraction (non—CMC 3×), in liver tissue (liver—3×) and in the cardiomyocyte fraction after the first round of selection (CMC—1×). (C) Variants that accumulated after three rounds of AAV peptide display library screening on hypertrophic CMCs. Shown are sequences of peptide inserts presented with amounts of more than 1.5%. *cap*, gene encoding capsid proteins (VP1-3) modified by peptide insertion as indicated, assembly activating protein (AAP) and membrane associated accessory protein (MAAP); *rep*, gene encoding AAV Rep proteins; *GFP*, gene encoding the enhanced green fluorescence protein (under control of a CMV promoter), CMC, cardiomyocytes; non-CMC, all cardiac cells except cardiomyocytes.

We performed an *in vivo* AAV2 peptide display library screen on hypertrophic cardiomyocytes induced by chronic left ventricular pressure overload after transverse aortic constriction (TAC) (Figure 1A). The library consisted of variants displaying peptides with a heptamer random sequence flanked by alanine linkers at I-587, i.e., between the capsid amino acid asparagine (N) 587 and arginine (R) 588 (VP1 numbering) in each of the 60 subunits of the AAV2 capsid, as previously described.^20^

Since AAV libraries using wild-type genomes with a modified *cap* open reading frame do not allow to determine the gene expression capacity of the variants during the screening procedure,^21^ we replaced for the second and third selection round the *rep* gene by an cytomegalovirus (CMV) promoter-driven-enhanced green fluorescent protein (*GFP*)-expressing transgene cassette and sorted by fluorescence-activated cell sorting (FACS) for GFP^+^ cardiomyocytes. However, only a very small fraction of cardiomyocytes showed sufficiently strong GFP expression for FACS-based detection (data not shown). Therefore, we isolated library genomes in all three rounds of selection from the whole cardiomyocyte population.

The enrichment of individual variants in the cardiomyocyte fraction after three rounds of selection (CMC-3x) was quantified by next-generation sequencing (NGS). Samples from the non-cardiomyocyte fraction (cardiac cells except cardiomyocyte, nonCMC-3x), liver tissue of the third round of selection (liver-3x), and the cardiomyocyte fraction from the first round of selection (CMC-1x) were included as off-target samples and a reference, respectively. *In silico* translation of the sequencing data revealed successful enrichment of 116 variants in CMC-3x in comparison with more than 12,000 variants in CMC-1x (Figure 1B). From these 116 variants, nearly one-half of the variants (53/116) showed cardiomyocyte-specific enrichment, i.e., the absence of respective sequences in liver-3x and non-CMC-3x samples.

By applying stringent selection criteria, including cardiomyocyte-specific detection, degree of enrichment, overall peptide charge, and amino acid composition (amino acid proline was favored owing to its unique structure) (Figure 1C), the capsid-modified variants AAV2-THGTPAD, AAV2-NLPGSGD, and AAV2-LPSRPSL were chosen for further characterization. Specifically, the variants AAV2-THGTPAD and AAV2-NLPGSGD were detected only in the cardiomyocyte-specific sample and showed a 1.85% (AAV2-THGTPAD) and 1.69% (AAV2-NLPGSGD) enrichment compared with the initial library. The third variant, AAV2-LPSRPSL, was by far the most enriched variant in CMC-3x (7.52%), but was also detected in the non-cardiomyocytes and liver samples.

### Novel variants differ in key features from AAV2

**Figure 2:**
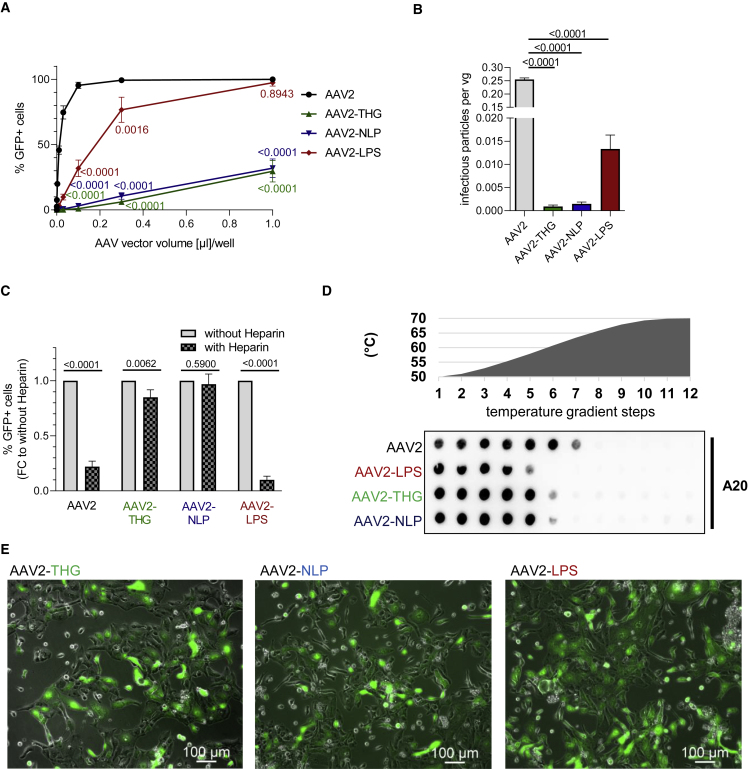
*In vitro* characterization of novel AAV capsid variants (A and B) Transduction efficiency of AAV vector variants and the parental AAV2 on non-cardiac cells *in vitro*. (A) Serial dilutions of respective vector preparations were used for the transduction of HEK293 cells in a 12-well format. Genomic titers of vectors used in this assay were in a comparable range. Transduction efficiency was determined by flow cytometry for GFP-positive cells 48 h after transduction (n = 3). (B) The ratio of infectious particles (on HEK293 cells) per vector genome was calculated based on transduction efficiencies obtained in (A) and the genomic titer measured by qRT-PCR. (C) Heparin competition assay. AAV vector variants and the parental AAV2 were pre-incubated with heparin or not. Transduction efficiency on HEK293 cells was determined by FACS for GFP positive cells 48 h after transduction (n = 3). (D) Thermal stability assay. AAV vector particles were subjected to different temperatures for 15 min followed by dot blotting using the A20 antibody for detection of assembled capsid proteins (intact AAV vector particles). (E) Performance of novel variants on cardiomyocyte cell model. Human iPSC-CMCs were transduced with AAV vectors expressing GFP with a vector particle-to-cell-ratio of 2 × 10^3^. GFP expression was assessed by fluorescence microscopy 7 days after transduction (n = 3). Data are means ± standard deviation. The p values were determined by one-way ANOVA with Dunnett’s multiple comparison with the parental serotype AAV2.

The three capsid variants and AAV2 were produced as viral vectors encoding for GFP driven by a human CMV promoter in a self-complementary vector genome conformation. After vector purification, we determined the infectivity of our variants on HEK293 cells to estimate a possible change in vector tropism. In line with the NGS profile, AAV2-THGTPAD and AAV2-NLPGSGD, the two variants detected exclusively in the cardiomyocyte target cell population, showed a severely decreased infectivity on HEK293 cells. In contrast, AAV2-LPSRPSL, the variant found enriched in target as well as off-target samples, showed a decreased infectivity compared with AAV2, but still transduced HEK293 cells to substantial amounts, particularly at higher particle-per-cell ratios (Figure 2A). This change in transduction efficiency on non-cardiac cells is perhaps even better revealed when correlating infectious and genomic titers of AAV2-THGTPAD and AAV2-NLPGSGD compared with AAV2 (and AAV2-LPSRPSL) (Figure 2B).

Based on the peptide sequence,^22^^,^^23^ as well as on the transduction profile in HEK293 cells, we assumed that AAV2-THGTPAD and AAV2-NLPGSGD likely transduce cells independent of HSPG, the primary or attachment receptor of AAV2.^23^ To assay whether our hypothesis is correct, we performed a heparin competition assay. The addition of heparin, the soluble analogue of HSPG, did not affect transduction by AAV2-NLPGSGD, suggesting that this variant transduces independent of HSPG. AAV2-THGTPAD efficiently transduced cells despite the presence of an access of heparin, but showed a slight decrease in comparison with the non-heparin-treated reference. In contrast, the presence of heparin strongly interfered with transduction by AAV2 and AAV2-LPSRPSL (Figure 2C).

Because lower capsid stability correlated with an improved transduction efficacy, likely owing to more efficient uncoating, i.e., a release of packaged viral vector genomes,^24^^,^^25^^,^^26^ we performed a thermal stability assay. As indicated, all three variants showed decreased capsid stability compared with AAV2 (Figure 2D). AAV2-LPSRPSL was the least stable variant, showing partial degradation of the capsid at 57.9°C. AAV2-THGTPAD and AAV2-NLPGSGD showed a comparable stability with partial capsid degradation starting at 60.7°C. In contrast, degradation of the AAV2 capsid only initiated at 63.4°C.

Finally, we tested our novel AAV variants on human induced pluripotent stem cell-derived cardiomyocytes (iPSC-CMC), confirming that all three variants not only accumulated in cardiomyocytes during *in vivo* selection in mice, but are also in principle capable of transducing human cardiomyocytes (Figure 2E).

### Novel AAV2-derived vector variants show a strong *in vivo* tropism for cardiomyocytes

**Figure 3:**
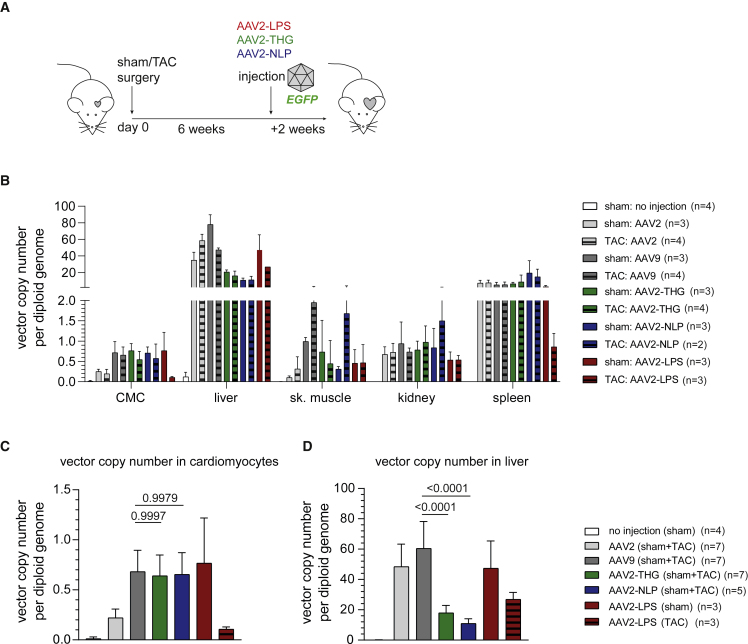
Vector copy number analysis (A) Indicated AAV vectors expressing *GFP* were injected in sham- and TAC-operated mice 6 weeks after sham and TAC surgery, respectively (note that each experimental group started with four mice each, but final group size differs owing to the loss of individual mice; n as number of mice is depicted for each group). Organs and cells were harvested 2 weeks after AAV vector injection for vector copy number and *GFP* expression analysis by qRT-PCR. (B) Overview of vector copy number analysis in different organs and cell type. (C and D) Comparison of the vector copy number in target cell types (cardiomyocytes [C]) and the main off-target organ (liver [D]). In (C) and (D), the mean vector copy numbers of pooled sham- and TAC-operated cohorts are shown for AAV2, AAV9, AAV2-THPTPAD, and AAV2-NLPGSGD (vectors that did not show noticeable differences between sham and TAC according to [B]). Data are means ± standard deviation. The p values were determined by one-way ANOVA with Dunnett’s multiple comparison with the current gold standard AAV9.

**Figure 4:**
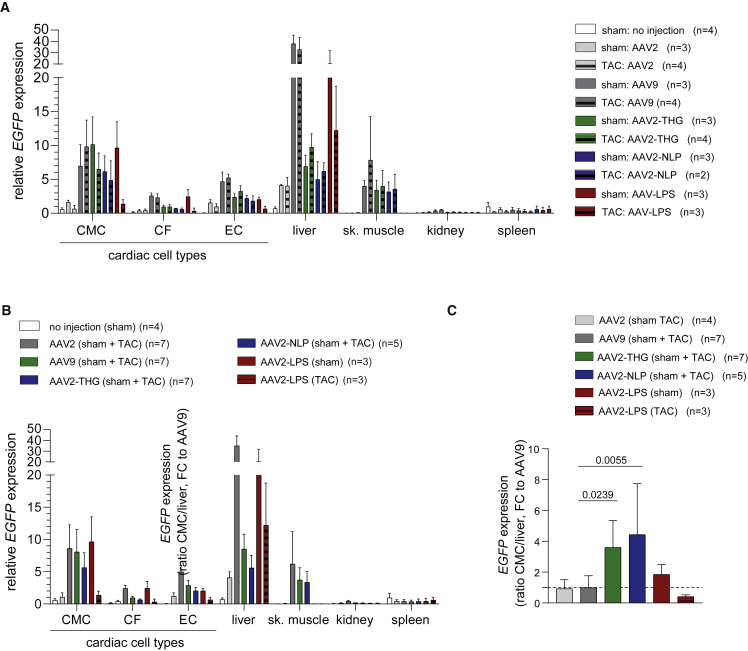
Transduction efficiency and specificity of AAV vector variants *in vivo* based on *GFP* expression AAV vectors (AAV2, AAV9, AAV2-THGTPAD, AAV2-NLPGSGD, and AAV2-LPSRPSL, expressing *GFP*) were injected in sham- and TAC-operated mice 6 weeks after sham or TAC surgery (note that each experimental group started with four mice each, but the final group size differs owing to the loss of individual mice; n as the number of mice is depicted for each group). *GFP* expression in cardiac cell types and different organs was analyzed by qRT-PCR 2 weeks after vector injection. (A) *GFP* expression analysis (separately for all different groups: sham or TAC with respective AAV vector variants) and (B) analysis based on pooled groups (sham + TAC mice, in case of AAV2, AAV9, AAV-THGTPAD, and AAV2-NLPGSGD—vectors that did not show noticeable differences between sham and TAC according to (B) (n = 3–7). (C) CMC to liver expression ratio normalized to AAV9 (n = 3–7). Data are means ± standard deviation. The p values were determined by one-way ANOVA with Dunnett’s multiple comparison with the current gold standard AAV9. GFP, self-complementary vector genome conformation encoding for enhanced green fluorescence protein; CF, cardiac fibroblasts; EC, cardiac endothelial cells; sk. muscle, skeletal muscle.

Based on the promising results obtained in human iPSC-CMC, we tested our novel variants *in vivo* in healthy (sham operated) mice or mice with cardiac hypertrophy (TAC operated). As a reference, we decided for AAV9 as frequently applied serotype in cardiac gene transfer and for AAV2 as the parental serotype. Vectors—again encoding for GFP—were injected into the tail vein of sham- or TAC-operated mice 6 weeks after surgery (Figure 3A). Vector biodistribution on DNA level (Figures 3B–3D), as well as transgene expression analysis (Figure 4), 2 weeks after AAV vector injection revealed a significantly improved tropism of AAV2-THGTPAD and AAV2-NLPGSGD for cardiomyocytes. Specifically, the novel variants clearly outperformed AAV2 in transduction of hypertrophic and physiologic cardiomyocytes (Figure 4), which correlated with significant improvement in vector copy numbers (Figure 3C). Vector copy numbers in sham as well as TAC-operated conditions reached values comparable with AAV9 (Figure 3C). AAV9, AAV2-THGTPAD, and AAV2-NLPGSGD showed a comparable transduction efficiency on hypertrophic and physiologic cardiomyocytes, respectively (Figure 4A). Furthermore, AAV2-THGTPAD reached transduction levels comparable with AAV9, being thereby slightly better than AAV2-NLPGSGD (Figure 4B). This gain in cardiomyocyte transduction efficiency is accompanied by a significantly decreased accumulation (Figure 3D), as well as transgene expression in liver tissue (Figure 4B). Notably, despite a significant accumulation of all vectors in the liver, which is the main off-target organ for AAV vectors, we observed a clear de-targeting from the liver with AAV2-THGTPAD and AAV2-NLPGSGD accumulating, approximately three times less compared with AAV2 and AAV9 (Figure 3B). Thus, in comparison with AAV9, AAV2-THGTPAD and AAV2-NLPGSGD present 3.6- and 4.4-fold improved cardiomyocyte-tropism, respectively, based on the target-to-noise expression ratios with cardiomyocytes defined as target and liver as off-target side (Figure 4C).

AAV2-LPSRPSL also transduced cardiomyocytes with greater efficiency than AAV2. Interestingly, AAV2-LPSRPSL showed—in contrast with the two other variants—a clear preference for healthy rather than hypertrophic cardiomyocytes (Figures 3B, 3C, 4A, and 4B). The levels of transduction of cardiomyocytes in sham-operated mice with AAV2-LPSRPSL were comparable with AAV9, while significantly lower levels were reached in TAC-operated mice. In line with the NGS pattern and the results obtained in HEK293 cells, AAV2-LPSRPSL showed no liver de-targeting in sham-operated animals. AAV2-THGTPAD and AAV2-NLPGSGD also transduced skeletal muscle comparable with AAV9 (Figures 4A and 4B). Regarding further cardiac cell types, cardiac fibroblasts and endothelial cells showed a very low but detectable transduction (Figures 4A and 4B).

### Enhanced nuclear accumulation of vector genomes and episome formation in the heart compared with liver tissue

**Figure 5:**
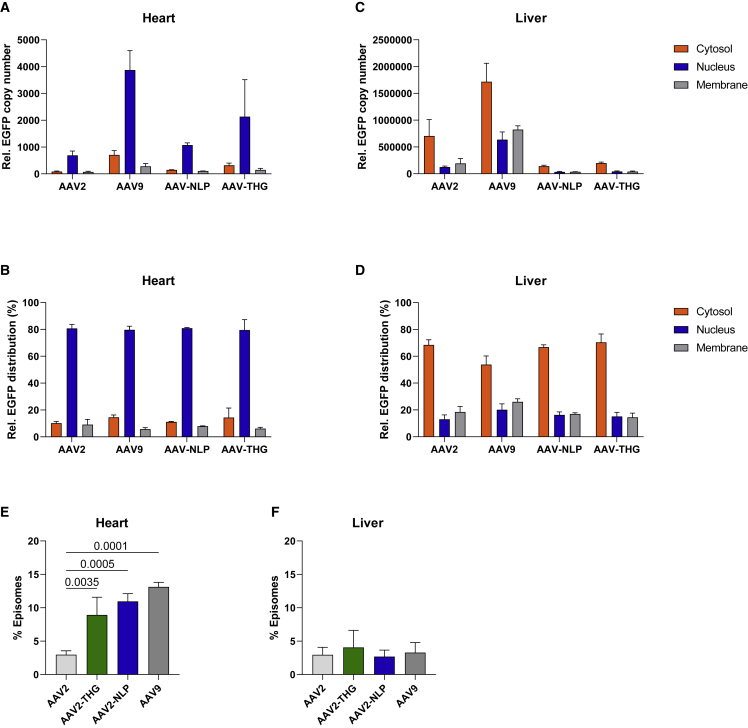
Subcellular vector distribution and episome formation in heart and liver tissue (A–D) Subcellular distribution of indicated AAV vector in different cell compartments (cytosol, nucleus, membrane; measured by qPCR and displayed as GFP transgene copy number concentration or as percentage distribution upon different cell fractions of heart (A and B) and liver tissue (C and D) from sham-operated mice 14 days after administration of respective vector variants. (E and F) Percentage proportion of vector genome formed as episomes in the nucleus of heart and liver tissue (n = 3). Data are means ± standard deviation. The p values were determined by one-way ANOVA with Dunnett’s multiple comparison with the parental serotype AAV2.

Since AAV entry barriers are known variables limiting target cell transduction, we determined the intracellular biodistribution of AAV2-THGTPAD and AAV2-NLPGSGD compared with AAV2 and AAV9 in tissue samples from the heart and liver obtained from sham-operated mice 2 weeks after injection. The heart nuclear fraction contained the highest level of vector genomes (Figures 5A and 5B). Interestingly, when analyzing liver tissue, vectors are mainly detected in the cytosolic fraction and thus were unable to contribute to transgene expression (Figures 5C and 5D).

The detection of vector genomes in the nuclear fractions does not correlate with templates available for transcription, since intact AAV vectors particles are known to enter the nucleus.^25^ We therefore performed an indirect uncoating assay using inherent resistance of episomes, the final vector genome conformation, to exonuclease degradation.^25^ DNA isolated from the nuclear fraction of heart and liver samples was, therefore, subjected to T5 exonuclease and compared with untreated controls. These analyses revealed a clear heart-related gain of function for AAV2-THGTPAD and AAV2-NLPGSGD (Figures 5E and 5F). Specifically, a significantly improved efficacy in episome formation was observed for both variants in heart tissue, while levels remained unchanged in liver tissue.

### Partial escape from the human pre-existing immunity and decreased *de novo* immunogenicity

**Figure 6:**
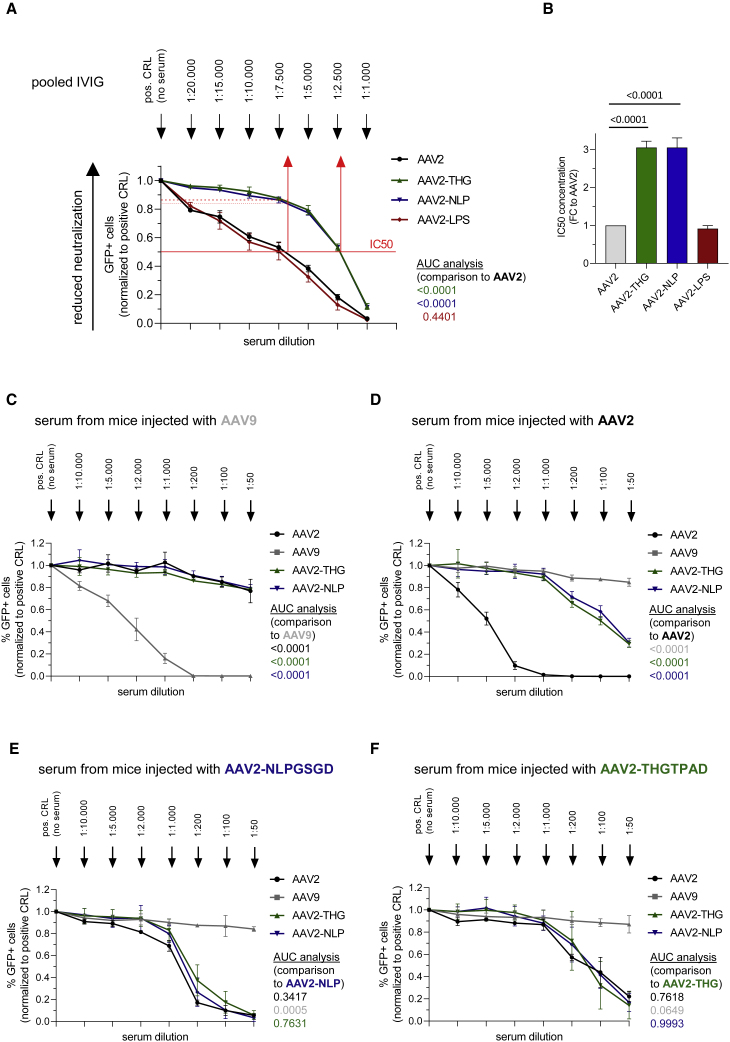
Serum neutralization assay (A) Neutralization assay of indicated AAV vectors by pre-incubation with human IVIG before transduction of HEK293 cells. For each AAV variant, as well as for each reference, the AAV vector amount, which induced 30%–40% of GFP-positive cells in the absence of IVIG (positive control) was chosen and incubated with indicated serum dilutions. The percentage of GFP-positive cells 48 h after transduction was analyzed by flow cytometry (IC_50_; n = 3 independent experiments). (B) IC_50_ was calculated as fold change to the parental AAV serotype AAV2. Neutralization assay of AAV variant as well as for references AAV vector variants on HEK293 cells by pre-incubation with pooled serum of mice which were previously injected with (C) AAV9, (D) AAV2, (E) AAV2-NLPGSGD and (F) AAV2-THGTPAD. For each AAV vector variant as well as for reference, the AAV vector amount which induced 30–50% of GFP-positive cells in the absence of any serum (positive control = without any serum) was chosen and incubated with indicated serum dilutions. The percentage of GFP-positive cells 48 h after transduction was analyzed by FACS. (n = 3 independent experiments; Data are means ± standard deviation. For area under the curve (AUC) analysis, the AUC was calculated separately for each of the individual experiment. p values were determined by one-way ANOVA with Dunnett’s multiple comparison).

Our targeting peptides are displayed at the tip of capsid protrusions, which are known immunogenic epitopes. To determine how this modification impacts on pre-existing humoral immune responses, we performed neutralization assays with human intravenous immunoglobulin (IVIG) serum covering thereby a broad range of antibodies. A challenge in this regard is the lack of a cell line that is equally well-transduced by our novel capsid variants and the parental serotype. Although we observed a lower sensitivity of AAV-THGTPAD and AAV-NLPGSGD compared with AAV2 for HEK293 (Figures 2A and 2B), we decided for HEK293 as an indicator cell, in line with Calcedo et al.^27^ We titered our vector preparations to identify a particle-per-cell ratio, which resulted in 30%–40% of GFP-positive cells in the absence of IVIG and used this ratio to determine the serum dilution that reduced our transduction efficiency by one-half. That is, we determined the half-maximal inhibitory concentration (IC_50_) (Figure 6A).

Both AAV-THGTPAD and AAV-NLPGSGD, but not AAV2-LPSRPSL, showed a significantly decreased sensitivity compared with AAV2 (Figure 6A). Serum dilution (1:7,500) close to the IC_50_ for AAV2 still allowed approximately 85% transduction (compared with the transduction efficiency without IVIG serum) by AAV2-THGTPAD and AAV2-NLPGSGD. Hence, both capsid-modified vector variants showed a 3.1-fold higher IC_50_ compared with the parental serotype AAV2 (Figure 6B).

Next, we studied the immunogenicity and cross-reactivity of our novel vector variants in more detail in the murine system. For this purpose, we used mouse serum collected from mice 2 weeks after AAV vector administration. Neutralization assays were performed by pre-incubating indicated vectors with these sera before transduction of HEK293 cells. Sera obtained from mice that had received AAV9 vectors neutralized AAV9 vector transduction in a concentration-dependent manner (Figure 6C). At very low serum dilutions, this serum only weakly decreased the transduction capacity of AAV2, which is in contrast to findings from Thwaite et al. investigating human sera (Figure 6C). The same holds true for the two novel AAV2-based variants (AAV2-THGTPAD and AAV2-NLPGSGD) (Figure 6C). Of note, however, in the presence of serum dilutions of up to 1:1,000 from mice previously injected with AAV2, which blocked transduction by AAV2 completely, AAV2-THGTPAD and AAV2-NLPGSGD retained their ability to transduce cells (Figure 6D). Moreover, serum of mice previously injected with AAV-NLPGSGD or AAV2-THGTPAD showed poor neutralization capacity, resulting in the neutralization of AAV2 and the novel vector variants only at very high serum concentrations (Figures 6E and 6F).

### Repeated AAV vector administration *in vivo*

**Figure 7:**
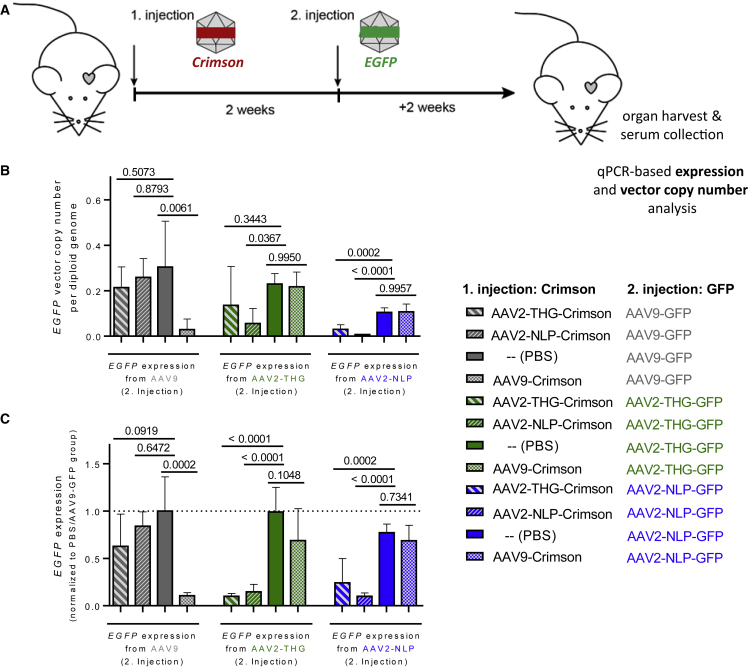
AAV2-THGTPAD and AAV2-NLPGSGD mediate efficient transgene expression in the heart despite previous AAV9 vector administration (A) AAV vectors encoding for *GFP* were injected 2 weeks after the administration of indicated AAV vectors delivering the *Crimson* transgene (4 × 10^10^ vector genomes or PBS as control). (B and C) Overview of vector copy number analysis (B) and expression analysis (C) of AAV *GFP* transgene encoding vector genomes (n = 5). Data are means ± standard deviation. The p values were determined by one-way ANOVA with Dunnett’s multiple comparison to the group which previously received PBS in the first injection. *Crimson* vector copy number and expression analysis are displayed in Figure S1.

We next tested whether a consecutive vector administration is possible mimicking condition of a vector re-administration. Therefore, mice were injected with AAV2-THGTPAD, AAV2-NLPGSGD, or AAV9 encoding for *Crimson*, a bright far-red fluorescent protein (Figure S1). Mice injected with PBS served as a positive control. After 2 weeks to allow the development of neutralizing antibodies, mice received a second vector administration with AAV particles encoding for *GFP* (Figure 7A). As expected, the greatest number of *GFP* vector copies/diploid genome and *GFP* mRNA expression in the heart was detected in cohorts that received PBS in the first round (e.g., without neutralizing antibodies) (Figures 7B and 7C). Comparable levels were achieved by AAV9-GFP in mice previously injected with AAV2-THGTPAD-Crimson or AAV2-NLPGSGD-Crimson. Similarly, mice initially injected with AAV9-Crimson showed no decrease in the *GFP* vector copies/diploid genome and *GFP* mRNA-expression mediated by a second vector administration with AAV2-THGTPAD or AAV2-NLPGSGD (Figures 7B and 7C). Using the same vector capsid for the consecutive administration showed—as expected—the lowest *GFP* vector copies/diploid genome and *GFP* mRNA expression. Switching one capsid variant for the other was also not sufficient to escape antibody neutralization.

Nevertheless, these results indicate that a second efficient transgene delivery and expression in cardiomyocytes *in vivo* is possible using our novel variants in combination with AAV9.

### Therapeutic efficacy and liver de-targeting of AAV2-THGTPAD and AAV2-NLPGSGD

**Figure 8:**
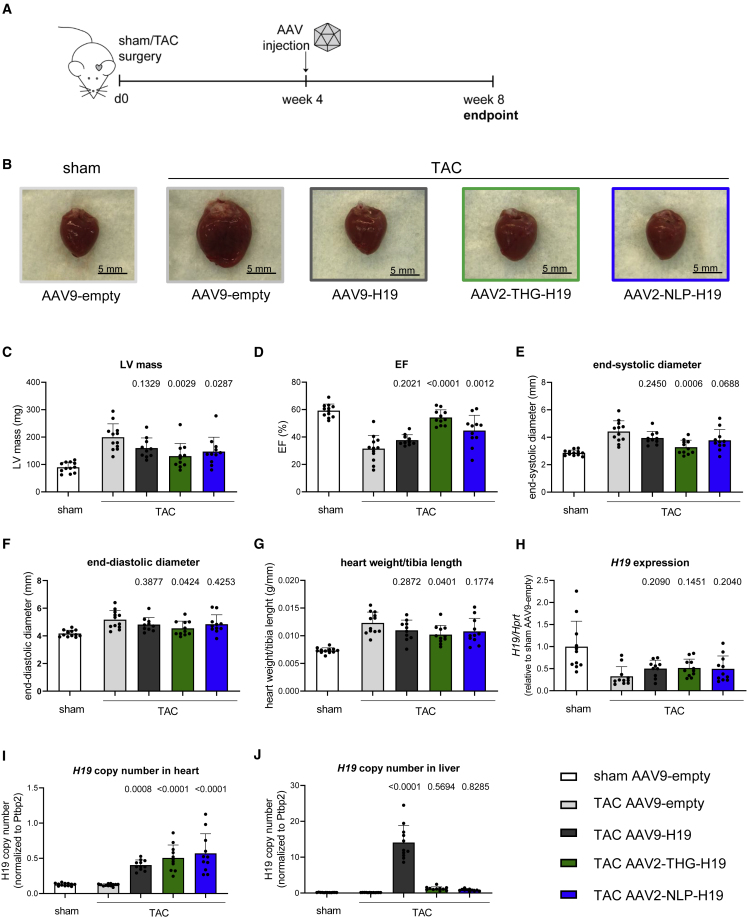
Improved gene therapeutic efficiency by AAV2-THGTPAD and AAV2-NLPGSGD delivering anti-hypertrophic lncRNA *H19* (A) Schematic representation of the experimental design. (B) Representative hearts of mice from the respective treatment groups. (C–F) Echocardiographic analysis at the end point of the study. (G) Heart weight to tibia length ratios. (H–J) (H) Expression levels of *H19* and (I, J) vector copy number analysis of *H19* in heart and liver tissue of sham- and TAC-operated mice treated with respective AAV variants delivering the lncRNA *H19* or control (AAV9-empty) (n = 10–12). Data are means ± standard deviation. The p values were determined by one-way ANOVA with Dunnett’s multiple comparison with the TAC AAV9-empty group.

Finally, we set out to test the novel AAV variants therapeutically. We have demonstrated recently that AAV9-based delivery of the long non-coding RNA (lncRNA) *H19* reverses pathological cardiac hypertrophy in the TAC mouse model.^29^ By analogy, we packaged *H19* into AAV9, AAV2-THGTPAD, and AAV2-NLPGSGD and injected mice 4 weeks after induction of TAC, i.e., when cardiac hypertrophy had already developed (Figure 8A). We hypothesized that, owing to the improved cardiomyocyte tropism low viral vector doses suffice. We therefore injected only 4 × 10^10^ vg/animal via the tail vein. The hearts of mice receiving *H19* gene therapy after TAC were less hypertrophied compared with the AAV9-empty group (Figure 8B). This was corroborated by functional echocardiography assessment data obtained 4 weeks after AAV treatment, showing significantly fewer left ventricular masses in the AAV2-THGTPAD-*H19* and AAV2-NLPGSGD-*H19* groups, whereas AAV9-*H19* only showed a non-significant trend (Figure 8C). In line, the left ventricular ejection fraction and cardiac dimensions for both AAV2-THGTPAD-*H19* and AAV2-NLPGSGD-*H19*, but not for AAV9-*H19*, were significantly rescued in comparison with the AAV9-empty control group (Figures 8D–8F; see Table S1 for a full set of echocardiography parameters). A lower heart weight to tibia length ratio was also observed (Figure 8G), but reaching significance only for AAV2-THGTPAD-*H19* treated mice. *H19* expression analysis after heart explantation showed the expected decrease in endogenous *H19* in cardiac hypertrophy.^29^ AAV2-THGTPAD-*H19*, AAV2-NLPGSGD-*H19*, and AAV9-*H19* treatment only partially rescued *H19* expression assessed in whole heart tissue (Figure 8H), presumably owing to the low vector dose in conjunction with the masking effects of the relatively greater endogenous *H19* expression in endothelial cells.^29^ Nevertheless, *H19* copy number analysis showed a strong increase for all variants compared with AAV9-empty controls (Figure 8I). Strikingly, while AAV9-*H19* strongly accumulated in the liver, *H19* copy numbers in AAV2-THGTPAD-*H19*, AAV2-NLPGSGD-*H19* treated mice were comparable with the sham and AAV9-empty control groups (Figure 8J), indicating an even more pronounced liver de-targeting at low vector dose (compare with Figures 3C and 3D).

## Discussion

In the last decades, AAV vectors emerged as the most promising *in vivo* gene therapy delivery vehicle with more than 160 human clinical trials and first market authorizations.^30^ However, transduction efficacy for therapeutic relevant target cells, including cardiomyocytes, is low. In addition, the broad tropism of natural AAV serotypes causes a “loss” of vector particles in off-target tissues. Both challenges are commonly addressed by high vector doses. In addition to increasing the costs of treatment, high vector doses are prone to induce immune responses. One example is onasemnogene abeparvovec (Zolgensma) (a recombinant AAV9-expressing the survival motor neuron [*SMN*] gene under the control of the chicken β-actin promotor) that is applied in vector doses of 1.1 × 10^14^ vg per kilogram body weight (kgBW) to transduce motorneurons in the central nervous sytem^2^^,^^31^ and is prone to induce acute serious liver injuries.^32^^,^^33^ For AAV8, another hepatotropic serotype, three fatalities owing to acute liver toxicity occurred in a human clinical trial on X-linked myotubular myopathy,^34^ a life-threating neuromuscular disease. Again, toxicities were associated with a very high vector dose of 3×10^14^ kgBW (no liver-related adverse events were observed at lower doses). These studies underscore the need to develop next-generation AAV vectors that exhibit improved target cell transduction efficacy combined with a decreased off-target activity.

As the capsid is the viral vector component interacting with host factors and surfaces, mediating entry into cells and being intracellular processed to finally release the viral vector genomes in the nucleus, most attempts to develop next generation AAV vectors address the capsid structure. Site-directed mutagenesis to change tyrosine, serine, threonine, or lysine residues—all of which are prone to become targets for tags recognized by the proteasomal system—to phenylalanine, valine, or glutamic acid, respectively, was demonstrated to improve transduction efficiency, both in cell culture and *in vivo.*^5^ AAV2i8, the next-generation AAV vector currently in human clinical trials for treating heart failure is considered a rational design-based approach to de-target AAV vectors from the liver and improve target cell transduction. A similar change in tropism was achieved by swapping 37 amino acids from the C-terminus of capsid proteins between AAV9 and AAV1 (reviewed in^14^). Particularly successful—especially in the absence of knowledge on receptors that might confer target cell selectivity—are high-throughput selection screens of AAV capsid libraries. The first example in the context of cardiac gene therapy was reported by Ying et al.,^35^ who used—like in our study—AAV2 and a peptide display library strategy in healthy mice and applying adenovirus co-infection. AAV9 has also been successfully explored as the basis of *in vivo* AAV peptide display library screenings.^36^ In addition to AAV peptide display library-based screenings, DNA shuffling library also showed promise with AAVM41 being one prominent example,^15^ as well as a random mutagenesis approach targeting surface-exposed region of the AAV capsid proteins.^37^

From the portfolio of capsid libraries, we decided for a AAV peptide display library approach to use the basal features of the parental serotype and to build on the knowledge of receptor binding as well as antibody recognizing epitopes. Specifically, AAV2 is considered a human AAV serotype and is particularly efficient in transducing cells of human origin. In addition, by site-directed mutagenesis or introduction of receptor targeting ligands—like we did when using I-587 for introducing our heptamer peptides of random sequence—the natural HSPG binding motif of AAV2 becomes destroyed, which, in combination with a target-selective ligand, was shown to result in next-generation AAV vectors for a precise and off-target free *in vivo* delivery after intravenous administration.^38^ Peptide insertion at this position—of course, depending on the peptide sequence, length, and structure—can also result in variants with immune escape features.^24^^,^^39^ A further interesting finding is that AAV capsid variants selected from AAV2 peptide display libraries maintain their improved target cell transduction efficacy and tropism, even when switching from mice (which were used for the high throughput selection screen) to larger animal models or human tissue (and further own unpublished own results).^24^

In contrast with our previous high-throughput selection screens, we here replaced the *rep* coding sequence for transgene expression cassette aiming to select our library based on GFP expression to identify variants, which are not only capable of entering cardiomyocytes, but are also processed in an efficient manner. However, although we injected the first and the second sub-libraries 2 weeks before harvesting to allow sufficient time for *GFP* transgene expression to be initiated and GFP protein to be accumulated, GFP expression detected by FACS was insufficient to reliably select transduced cells. Considering our subsequent NGS analysis and the results obtained for selected variants as individual vectors, this failure was surprising in retrospect. Specifically, AAV2-THPTPAD, AAV2-NLPGSGD, and AAV-LPSRPSL represented approximately 10% of all sequencing reads of the third and final round of selection and do express from vector genomes as indicated in Figure 4. Thus, the most plausible explanation is that the GFP expression during the selection process was in general too low for FACS-based detection.

The change in tropism of AAV2-THGTPAD and AAV2-NLPGSGD, which was evident already when performing transducing titer assays on HEK293, was clearly demonstrated *in vivo* at two different vector doses, using different transgenes and TAC- as well sham-operated animals. Specifically, on DNA and expression level we observed a de-targeting from liver and—compared with AAV2—an improved transduction of cardiomyocytes. The higher vector copy numbers of all variants compared to AAV2 already argued for improved cell entry. The subcellular biodistribution then revealed that, in addition, our targeting vectors are transported efficiently to the nucleus. As indicated by our indirect uncoating assay, they are more efficiently processed toward reaching the AAV episome conformation, which is considered as a long-term persisting and transcriptionally active AAV vector configuration.

Interestingly, the variant LPSRPSL, which was enriched in cardiomyocytes, but also detected in non-cardiomyocytes and liver tissue in our screening, was also found enriched in a peptide display library on human monocyte-derived immature dendritic cells,^25^ corroborating that this variant might use a receptor that is shared among different cell types. Receptor expression seems to differ in hypertrophic versus physiological cardiomyocytes, because fewer AAV2-LPSRPSL were detected in TAC- versus sham-operated mice, a difference in line with the lower expression in TAC- compared with sham-operated mice.

For AAV2-THPTPAD and AAV2-NLPGSGD, we did not observe such clear differences in vector copy number and expression analysis between TAC- and sham-operated mice, implying that these variants use cell surface receptors that are not differentially regulated upon TAC-induced hypertrophy. Which (glyco-) protein structures on the surface of cardiomyocyte are crucial for the uptake of AAV2-THPTPAD and AAV2-NLPGSGD, however, is currently unknown.

In terms of translation of our findings to a human context and a potential application of these vectors in clinical trials in the future, result on human iPSC-derived cardiomyocytes are promising; they argue that our variants, albeit selected in mice, are transducing cells of human origin and, thus, likely possess a cross-species activity.

In line with our previous report,^23^ the insertion of negatively charged 7-mer peptides (THPTPAD and NLPGSGD) into I-587 destroyed not only the common interaction of AAV2 with its primary receptor HSPG, but also avoided that; owing to the peptide insertion, the variant re-gains the ability to bind this proteoglycan. Thus, heparin competition during transduction was ineffective (Figure 3C). This is in contrast with the insertion of positively charged peptides (as it is the case for LPSRPSL), which is generally associated with retrieving HSPG binding capacity and liver tropism.^22^^,^^40^

The insertion of peptides at I-587 also affects B cell epitopes,^39^ and thus is expected—depending on the antibody panel present in a distinct sera—to enable transduction in the presence of neutralizing antibodies. We here assayed IVIG as well a panel of mouse sera. IVIG commonly leads to the neutralization of AAV vectors already in low serum concentrations.^41^ This reflects the prevalence of neutralizing antibodies and indicates one of the main challenges of AAV vector-based gene therapy; a major proportion of patients have to be excluded from trials owing to pre-existing neutralizing antibodies against naturally occurring AAV serotypes.^10^^,^^42^ AAV2-THPTPAD and AAV2-NLPGSGD, however, showed significantly lower sensitivity to IVIG compared with AAV2 or AAV2-LPSRPSL, although we must admit that we had to perform these assays on HEK293 cells as no cell line efficiently transduced by all three variants and AAV2 is available. Undoubtedly, an interesting immune response-related feature of our novel variants is the lower anti-AAV activity of sera from mice immunized with AAV2-THPTPAD and AAV2-NLPGSGD, a feature that we are interested in to investigate further. Furthermore, our *in vivo* neutralization experiments revealed that the administration of AAV2-THPTPAD or AAV2-NLPGSGD does not lead to immunity against AAV9 and *vice vera*, potentially enabling their consecutive use if necessary. This observation is of importance; Thwaite et al.^28^ reported that human sera that neutralizes AAV9 also neutralizes AAV2, the parental serotype of our novel capsid variants. However, this experiment also revealed that—at least in the setting we tested—antibodies elicited against our vectors, including the novel variants, are potent in impairing sufficient transduction after re-application of the same vector. Whether the situation is different when a second administration is performed at a later time point, i.e., in the presence of lower antibody titers remains to be elucidated.

Finally, our study demonstrated a strong therapeutic potential for the treatment of cardiac disease using the variants AAV2-THPTPAD and AAV2-NLPGSGD equipped with the anti-hypertrophic lncRNA *H19*. The administration of a 14-times lower vector dose compared with our previous study using AAV9-*H19* vectors^29^ led to a significant improvement in cardiac function and rescue from the cardiac hypertrophy phenotype. Importantly, this therapeutic setup re-confirmed that the novel variants AAV2-THPTPAD and AAV2-NLPGSGD are efficiently re-targeted to cardiomyocytes, showing a similar or even higher transduction efficiency in mediating an enhanced therapeutic benefit compared with the gold standard AAV9. In addition, AAV2-THPTPAD-*H19* and AAV2-NLPGSGD-*H19* were drastically de-targeted from the liver, which underlines their highly relevant clinical potential.

In summary, by applying a state-of-the-art *in vivo* screening of an AAV2-based peptide display library, we identified two highly cardiomyocyte-specific capsid variants. These novel variants mediated significant therapeutic effects in a heart failure mouse model at low vector doses, along with significant liver de-targeting and immune escape. Therefore, these variants represent highly relevant gene therapy vectors for the treatment of cardiac disease, which strongly warrants further development toward clinical application.

## Material and methods

### Cell lines

The human embryonic kidney cell line HEK293 (purchased from German Collection of Microorganisms and Cell Cultures GmbH, Braunschweig) was maintained in DMEM supplemented with 10% fetal bovine serum (FBS) and 1% penicillin-streptomycin (P/S) and cultured at 37°C and 5% CO_2_.

iPSC (stem cell line: hHSC_Iso4_ADCF_SeV-iPS2 [Phoenix]) were kindly provided by Robert Zweigert (MHH) and were generated from human cord blood CD34^+^ cells as described by Haase et al.^43^ Cardiomyocytes were derived from iPSCs using a protocol based on modulation of the Wnt signaling pathway as described by Lian et al.^44^

### Animal experiments

All animal experiments were performed in accordance with the guidelines and regulations of the Lower Saxony State Office for Consumer Production and Food Safety (LAVES, Nds. Landesamt für Verbraucherschutz und Lebensmittelsicherheit, animal license number 16/2146). Mice were randomly assigned to treatment groups. To induce cardiac pressure overload, male C57BL/6N mice (6–9 weeks old) were subjected to TAC as described previously,^45^ using a 26G needle. Aortic stenosis by TAC was confirmed by echocardiography before AAV injection. Sham-operated animals underwent the same procedure without constriction of the aorta. In all experiments, mice received AAV library or vector solution by tail vein injection. In the first round of selection, 8 × 10^11^ library genomes per animal were injected, while the dose was decreased for the second and third rounds to 1 × 10^11^ library genomes per animal. For the biodistribution experiment, 1 × 10^11^ vector genomes per animal were injected, while in the repeated AAV injection study mice received 4 × 10^10^ vector genomes of the first vector (delivering the *Crimson* transgene) and 4 × 10^10^ vector genomes of the second (*GFP*-encoding) vector. Also in the therapeutic proof-of-concept study, 4 × 10^10^ vector genomes were injected per mouse. Therapeutic overexpression of lncRNA *H19* in the TAC mouse model was performed as described recently^29^ with the exception that the vector construct contained a CMV promoter in front of the murine *H19* sequence.

Echocardiographic measurements were performed at baseline, before AAV injections, and at the experimental end point with a Vevo 2100 system (VisualSonics Inc.).

### Cardiac cell fractionation

Adult mouse cardiomyocytes were isolated using the retrograde perfusion method as previously described,^46^ with slight modifications. Hearts were cannulated through the aorta and retrograde flushed with prewarmed perfusion buffer (113 mM NaCl, 4.7 mM KCl, 0.6 mM KH_2_PO_4_, 0.6 mM Na_2_HPO_4_, 1.2 mM MgSO_4_-7H_2_O, 0.032 mM phenol red, 12 mM NaHCO_3_, 10 mM KHCO_3_, 10 mM HEPES, 30 mM taurine, 0.1% glucose, 10 mM 2,3-butanedionemonoxime) for 3 min within the mouse, then removed from the mouse and perfused for additional 3 min with a constant flow rate of 4 mL/min. The buffer was switched to pre-warmed digestion buffer (perfusion buffer supplemented with 700 U/mL collagenase II (Worthington Biochemical Cooperation) and 12.5 μM CaCl_2_) for 10 min. The atria were removed, and the ventricles were dissociated mechanically by cutting the tissue in 2.5 mL warm digestion buffer and shearing through a 1 mL syringe. Collagenase II digestion was stopped by adding 2.5 mL stop buffer (perfusion buffer containing 10% FBS and 12.5 μM CaCl_2_), followed by tissue shearing through a 1-mL syringe for another 2 min. The obtained cell suspension was filtered through a 100-μm cell strainer and the filter was washed once with 1–2 mL AMCF medium (MEM supplemented with 10% FBS, 2 ng/mL vitamin B12, 4.2 mM NaHCO_3_, 100 U/mL penicillin, 100 μg/mL streptomycin, pH 7.3). Cardiomyocytes were separated from other cardiac cell types by sedimentation for 10 min at room temperature, washed with PBS, pelleted at 3,000 rpm for 5 min and snap frozen in liquid nitrogen. The remaining supernatant was centrifuged for 3 min at 30×*g* at room temperature to remove residual cardiomyocytes. The remaining supernatant containing the non-myocyte fraction was centrifuged at 430×*g* for 5 min at room temperature, followed by dissolving the obtained cell pellet in AMCF medium (MEM supplemented with 10% FBS, 2 ng/mL vitamin B_12_, 4.2 mM NaHCO_3_, 100 U/mL penicillin, 100 μg/mL streptomycin, pH 7.3) and pre-plated on a 10 cm Petri dish in a 1% CO_2_ incubator for 1 h. The attached cells (cardiac fibroblasts) were washed with PBS twice, then 2 mL PBS were added to the dish and cells were harvested with a cell scraper, centrifuged at 900×*g* for 5 min at 4°C. The pellet was frozen in liquid nitrogen and stored at −80°C. The supernatant of the pre-plating step, containing the non-myocyte, non-fibroblast fraction, was centrifuged at 430×*g* for 5 min at 4°C. The resulting cell pellet was resuspended in 80 μL MACS buffer (MACS bovine serum albumin stock solution diluted 1:20 in auto-MACS rinsing solution, both from Miltenyi Biotec) mixed with 20 μL CD146 MACS beads (Miltenyi Biotec) and incubated for 15 min at 4°C. Afterward, 2 mL of MACS buffer was added, the cell suspension was mixed thoroughly and centrifuged for 5 min at 430×*g* at 4°C. The cell pellet was resuspended in MACS buffer and transferred to a pre-washed MACS separating column. After three washing steps with MACS buffer, the separation columns were removed from the magnetic field and cardiac endothelial cells were collected by rinsing the column three times with 500 μL MACS buffer and centrifuged at 900×*g* for 5 min at 4°C. The cell pellet was frozen in liquid nitrogen and stored at −80°C.

### Subcellular fractionation and uncoating assay

Frozen tissue samples from sham-operated mice injected with indicated AAV vectors were used for the subcellular fractionation (50 mg liver tissue and 40 mg heart tissue) using the Subcellular Protein Fractionation Kit for Cultured Cells (ThermoFisher Scientific, #78840). Tissue samples were then put into Precellys tubes with beads (6 × 2.8 mm beads per tube) together with 500 μL CEB buffer. The tissue was homogenized twice for 10 s at 5,000 rpm with the Precellys homogenizer. The lysate was then applied to a cell strainer and was directly transferred into the NcF tube. Subsequently, the nuclear fraction was separated by centrifugation for 5 min at 500×*g*, which remained on ice until further processing. The supernatant was transferred into a new tube (MbF) and was again centrifuged for 10 min at 17,000×*g*. The supernatant then contained the cytosolic fraction, which was kept on ice. Next, the pellet was resuspended with 500 μL MEB buffer and was combined with the pellet of the nuclear fraction. After centrifuging for 5 min at 3,000×*g*, the supernatant was transferred into a new tube containing the membrane fraction. The pellet was resuspended with 500 μL NEB buffer, which represented the nuclear fraction. Afterward, the samples were stored at −80°C before DNA isolation with the Qiagen DNeasy Blood & Tissue Kit. Here, the DNA was eluted with 100 μL ddH_2_O and 2 μL were used for qRT-PCR quantification of *GFP* expression levels by comparing it to a *GFP* plasmid standard. Isolated DNA from the nuclear fractions were either digested with T5 exonuclease or with mock treatment.^25^

### AAV peptide display library selection and AAV vector production

AAV peptide display library production as well as AAV vector production were performed in HEK293. The initial library had been subjected to a genotype/phenotype coupling step as described previously,^25^ while the expression sub-libraries were genotype/phenotype coupled by decreasing the plasmids transferred per cells during vector production (150 ng library plasmid pool per 15-cm plate). Cell lysates were purified by iodixanol gradient purification.^25^ Genomic AAV (vector) particle titers were determined by qPCR (LightCycler System, Roche Diagnostics) using *GFP*- or ITR-specific primers.

AAV2 capsid variants were selected by screening an AAV2 peptide display library on hypertrophic cardiomyocytes *in vivo*. Three rounds of selection were performed in TAC-operated mice. Material obtained from 2–4 mice was pooled after each selection round before cloning the subsequent sub-library^26^ or NGS analysis. Compared with the initial library published by Perabo et al.,^20^ the AAV *rep* gene was replaced by a CMV-GFP expression cassette for application of reporter sub-libraries in the second and third selection rounds (AAV *rep* was provided in *trans* during AAV vector production). In the first round, the initial library was injected in mice 53 days after TAC surgery. Three days later, fractionation of the cardiac cells was performed. To allow efficient *GFP* expression, AAV vector sub-libraries of the second and third selection rounds were injected 42 days after TAC surgery and cardiomyocytes, non-myocyte cells as well as liver tissue were harvested 2 weeks after AAV sub-library injection. FACS (MoFlo XDP Cell Sorter, Beckman Coulter, using a 150-μm nozzle) for GFP^+^ cardiomyocytes was performed 2 and 4 weeks after AAV sub-library injection. After three *in vivo* selection rounds, library DNA in the cardiomyocyte fraction (CMC—3×), in the non-myocyte cell fraction (non—CMC-3×) and in liver tissue (liver—3×) after the third round of selection as well as in the cardiomyocyte fraction after the first round of selection (CMC—1×) was analyzed by NGS. For NGS library construction, CMC and non-CMC DNA were applied to targeted PCR using the GeneRead DNAseq Panel PCR Kit (Qiagen) and a primer set (5-ʹTGGAATCTTTGCCCAGATGG-3′, 5′-ACAACCAATCCCGTGGCTAC-3′) flanking the variable region of the *cap* gene. After amplicon purification, NGS libraries were then prepared by means of the GeneRead DNA Library I Amp Kit (Qiagen) according to the manufacturer′s user guide, followed by sequencing on the MiSeq platform (Illumina, Inc.) using the v2 chemistry as recommended by the manufacturer.

Based on the NGS results, candidates were picked as described in the Results section and respective nucleotide sequences encoding these peptides were cloned into the *cap* gene of pRC′99^47^ to obtain helper plasmids for producing capsid variants as vectors.

### RNA isolation

Total RNA from cardiac cell fractions and mouse organ tissues was isolated using the miRNeasy Mini Kit (Qiagen) according to the manufacturer`s protocol. Mouse tissue samples and cardiomyocyte cell pellets where homogenized using the Precellys24 homogenizer after adding Qiazol Lysis Reagent to the sample. DNA digest on column was included using the RNase-Free DNase Set (Qiagen) (in the *H19* therapeutic overexpression study, DNase digestion was performed only after RNA elution before reverse transcription). RNA was eluted in H_2_O. RNA concentration and purity were determined by measuring the sample absorption at 260 nm and 280 nm using the Synergy HT Reader (BioTek).

### Transgene expression analysis

Reverse transcription of total RNA was performed using the Biozym cDNA Synthesis Kit (Biozym) according to the manufacturer`s instruction. In case of organ tissue samples, a second DNase digestion was performed directly before reverse transcription by incubating 500 ng total RNA with 0.684 μL DNase (1:10 dilution, RNase-Free DNase Set [Qiagen]), 1.15 μL RDD buffer (RNase-Free DNase Set [Qiagen]), and 0.144 μL RNasin Ribocluclease Inhibitor (Promega) in a total volume of 11.5 μL for 30 min at 37°C. The reaction was stopped by adding 0.23 μL 62.5 mM EDTA and incubation for 5 min at 65°C. RNA dilutions of 11.5–11.73 μL containing 65–500 ng RNA (with or without second DNase digest) were reversed transcribed using 4 μL 5× cDNA synthesis buffer, 2 μL dNTP Mix (10 mM each), 1 μL hexamer primer (25 μM), 0.5 μL RNase inhibitor (40 U/μL), and 1 μL reverse transcriptase. The reaction mix was incubated at 30°C for 10 min, followed by 60 min at 55°C; finally, the enzyme was heat inactivated at 99°C for 5 min. Before qRT-PCR, cDNA samples were diluted with two volumes of nuclease-free H_2_O (1:3 dilution). qRT-PCR measurements were performed in a 384-well format using the iQ SYBR Green Supermix (BioRad) according to the manufacturer’s instructions and was run on a Viia 7 Real-Time PCR System (ThermoFisher Scientific). *GFP* expression (*GFP* forward primer: 5′-CACAACGTCTATATCATGGC-3′; *GFP* reverse primer: 5′-TGTGATCGCGCTTCTC-3′) in cardiac cell fractions and murine organs was analyzed by the relative standard method. A 1:5 dilution series of pooled *GFP* expressing samples was included in all qRT-PCR measurements to obtain relative standard values for all samples. *Tbp* (*Tbp* forward primer: 5′-TGGAATTGTACCGCAGCTTCA-3′; *Tbp* reverse primer: 5′-CTGCAGCAAATCGCTTGGGA-3′) was used for normalization on RNA input in the *GFP* expression analysis. *H19* expression (*H19* forward primer: 5′-CTCCTCCCCCTACCTTGAAC-3′; *H19* reverse primer: 5′-CCTTGGAGCAGATTCCTGAG-3′) was analyzed by the ΔΔCt method using *Hprt* (*Hprt* forward primer: 5′-GCGTCGTGATTAGCGATGAT-3′; *Hprt* reverse primer: 5′-TCCTTCATGACATCTCGAGCA-3′) for normalization on RNA input.

### DNA isolation

DNA of cardiomyocytes and organ tissue was isolated using the DNeasy Blood and Tissue Kit (Qiagen) (the optional RNase A digestion step was included). DNA was eluted in 35 μL H_2_O. DNA concentration and purity were determined by measuring the sample absorption at 260 nm, 280 nm, and 320 nm using the Synergy HT Reader (BioTek).

### Vector copy number analysis

AAV vector copy number was determined via relative qRT-PCR quantification (QuantStudio Real-Time PCR System, ThermoFisher Scientific). In detail, the absolute gene copy number of *GFP* or *H19* was normalized to the absolute gene copy number of the reference gene *Ptbp2* (polypyrimidine tract binding protein 2; two copies per diploid genome). Multiplex TaqMan probe-based qPCR detection was performed in a 384-well format using the TaqMan Fast Advance Master Mix (ThermoFisher Scientific), the TaqMan Copy Number Assay for *GFP* (Mr00660654_cn, ThermoFisher Scientific; FAM fluorescent labeled) or a TaqMan assay for *H19* (Mm01156721_g1, ThermoFisher Scientific), 330 nM *Ptbp2* primer (forward: TCTCCATTCCCTATGTTCATGC, reverse: GTTCCCGCAGAATGGTGAGGTG) and 150 nM JOE-fluorescent labeled *Ptbp2* probe (5ʹ [JOE]-ATGTTCCTCGGACCAACTTG-[BHQ1] 3′). A standard curve of linearized plasmid (5 × 10^5^, 5 × 10^4,^ 5 × 10^3,^ 5 × 10^2^, 5 × 10^1^ molecules/μL) was used for quantification.

### *In vitro* transduction of human iPSC-derived cardiomyocytes

Differentiated iPSC-derived cardiomyocytes (1 × 10^6^ cells/well seeded in a 12-well plate format) were transduced with respective AAV vector variants in a self-complementary genome conformation expressing *GFP* from the CMV promoter with a vector particle-to-cell-ratio of 2 × 10^3^. GFP expression was assessed by fluorescence microscopy (Eclipse Ci, Nikon) 7 days after transduction.

### Transduction efficiency and heparin competition assay on HEK293 cells

We seeded 3.5 × 10^5^ cells per well (12-well format) 24 h before transduction in culture medium (DMEM 10% FBS 1% P/S). Cells were incubated with a serial dilution of AAV vector preparation. For heparin competition, cells were transduced with the lowest dilution (i.e., the highest concentration of the vector) in the presence of 425 IU/mL heparin. Percentage of transgene-expressing cells was determined by flow cytometry 48 h later (FACSCalibur, BD Bioscience).

### Capsid thermal stability assay

Capsid stability of the AAV vectors was assessed as previously described.^25^ Briefly, 2 × 10^9^ vg of respective AAV vectors were exposed to indicated temperatures (50.0°C, 51.0°C, 52.9°C, 55.3°C, 57.9°C, 60.7°C, 63.4°C, 65.8°C, 67.9°C, 69.3°C, 69.9°C, and 70°C), subsequently diluted in PBS-Mg/K and transferred to a nitrocellulose membrane using a vacuum blotter. Intact capsids were detected by an A20 antibody^48^ and a horseradish peroxidase-conjugated secondary antibody (Sigma, 1/10,000 dilution).

### Neutralization assay

Briefly, 3.5 × 10^5^ HEK293 cells per well (12-well format) were seeded 24 h before transduction. Vectors were used in an amount resulting to approximately 30%–40% of transgene expressing cells 48 h after transduction in the absence of neutralization antibodies. Vectors diluted in DMEM medium (supplemented with 10% FBS and 1% P/S) were incubated for 1 h at room temperature with different serum dilutions, either human IVIG serum (Intratect 50 g/L, Biotest Pharma GmbH; pre-diluted in 50% glycerol) or mouse serum obtained from mice that were previously injected with one of the vector variants expressing CMV-*GFP* in a self-complementary genome conformation. The mixture of vector and serum diluted in medium was added onto the cells and incubated for 48 h at 37°C and 5% CO_2_. Transduction efficiency was analyzed by flow cytometry (FACSCalibur, BD Bioscience) by determining the percentage of GFP-positive cells.

### Statistical analysis

Data are presented as the mean ± standard deviation. Statistical analyses were carried out using GraphPad Prism Version 6 and 8 (GraphPad Software). Normal distribution of data was assumed and for comparison of three or more groups, one-way ANOVA followed by Dunnett’s post hoc test was applied. A p value of 0.05 or less was considered significant in all experiments.

### Data availability

All data are included in this article. Raw data can be made available upon reasonable request.

## Supplemental information

Document S1. Figure S1 and Table S1

Document S2. Article plus supplemental information
