# Supplementary material for: AAV capsid engineering identified two novel variants with improved in vivo tropism for cardiomyocytes
Source: Mol Ther. 2022 Jul 9;30(12):3601–18. doi: 10.1016/j.ymthe.2022.07.003 (PMC9734024; doi:10.1016/j.ymthe.2022.07.003)
Supplement: Document S1. Figure S1 and Table S1 [file mmc1.pdf]

YMTHE, Volume *30*

Supplemental Information

AAV capsid engineering identified two novel variants with improved *in vivo* tropism for cardiomyocytes

Laura Rode, Christian Bär, Sonja Groß, Axel Rossi, Nadja Meumann, Janika Viereck, Naisam Abbas, Ke Xiao, Isabelle Riedel, Anika Gietz, Karina Zimmer, Margarete Odenthal, Hildegard Büning, and Thomas Thum

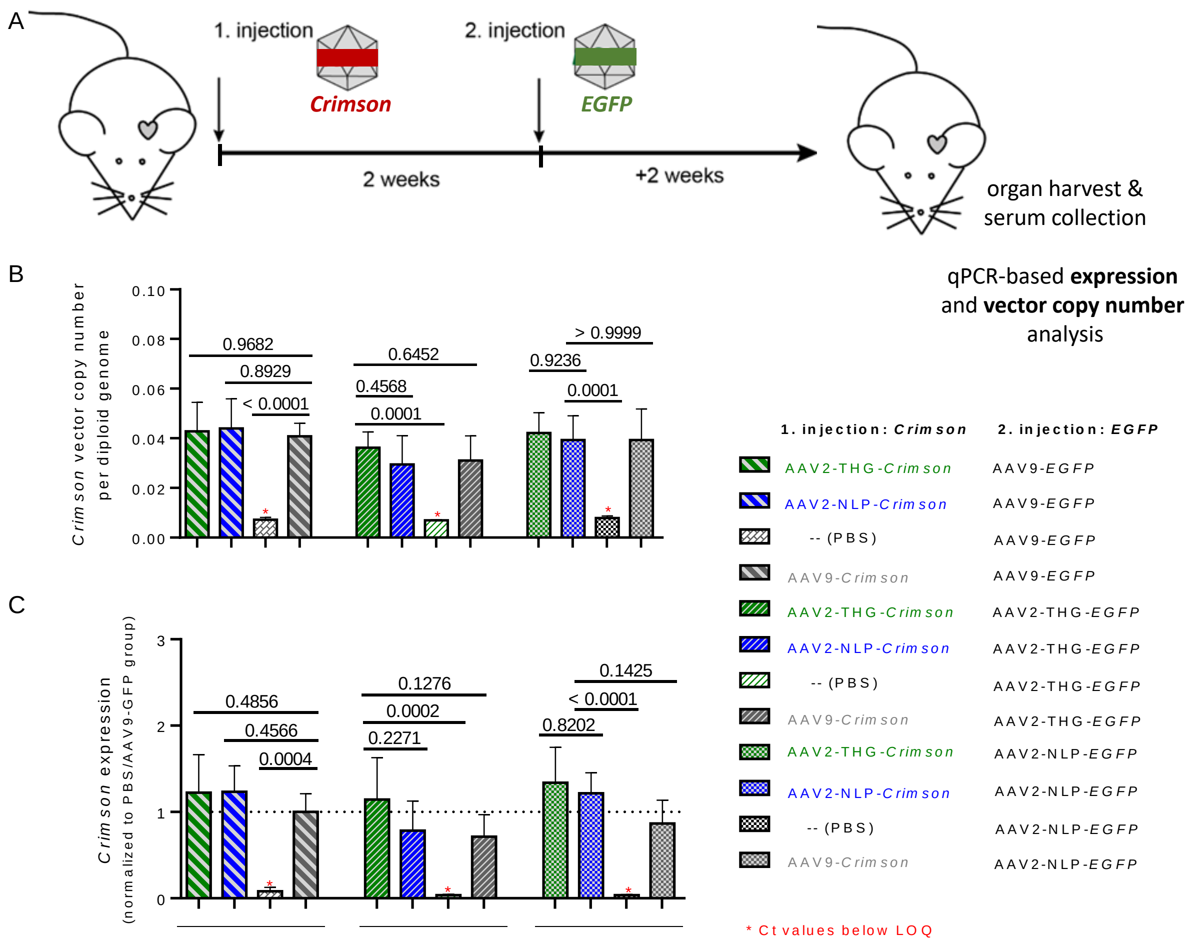

Supplemental Fig. 1: *Crimson* vector copy number and expression analysis.

(A) AAV vectors delivering *GFP* were injected two weeks after a previous AAV vector injection of different AAV variants delivering the *Crimson* transgene ($4x10^{10}$ vector genomes or PBS as control). (B+C) Overview of vector copy number analysis (B) and expression analysis (C) from the first injected AAV vector (*Crimson* transgene) showing comparable vector copy numbers and expression levels, respectively, in all groups receiving the *Crimson* transgene by AAV-mediated delivery. n=5, data are means ± SD. P-values were determined by one-way ANOVA with Dunnett’s multiple comparison to the group which received the same AAV capsid variant (AAV2-THG, AAV2-NLP, AAV9) in the first and second injection. (LOQ: limit of quantification)

Supplemental Table 1: Echocardiographic analysis of sham and TAC mice after therapeutic overexpression of lncRNA *H19* using AAV9, AAV2-THGTPAD and AAV2-NLPGSGD.

	sham	TAC			
	AAV9-empty	AAV9-empty	AAV9-H19	AAV2-THG-H19	AAV2-NLP-H19
**LV ejection fraction (%)**	59.2 ± 4.8	31.5 ± 9.7	35.6 ± 8	54.2 ± 5.6 ***	44.7 ± 11.1 *
**Fractional shortening (%)**	31.2 ± 3.4	15.1 ± 5.1	17.2 ± 4	28.1 ± 3.5 ***	22.5 ± 6.4 *
**Cardiac output (µl/min)**	20.9 ± 5	18.5 ± 5.5	18.3 ± 7.4	24.7 ± 6.9	23.8 ± 7.4
**LV mass (mg)**	89.8 ± 18.8	199.6 ± 48.7	167.7 ± 44.1	130.4 ± 46.5 **	147.3 ± 52.4 **
**End-diastolic volume (µl)**	77.6 ± 10.7	130.7 ± 38.8	117.9 ± 37.6	96.2 ± 26.7	112.6 ± 38.3
**End-systolic volume (µl)**	31.6 ± 5.4	92.2 ± 38.2	78.1 ± 35.9	45.1 ± 17 **	64.9 ± 33.5
**Stroke volume (µl)**	46 ± 7.8	38.5 ± 8.3	39.8 ± 8.9	51.1 ± 10.4 **	47.7 ± 11.4
**End-diastolic diameter (mm)**	4.2 ± 0.2	5.2 ± 0.7	4.9 ± 0.6	4.5 ± 0.5 *	4.8 ± 0.7
**End-systolic diameter (mm)**	2.9 ± 0.2	4.4 ± 0.8	4.1 ± 0.7	3.3 ± 0.5 ***	3.8 ± 0.8
**End-diastolic anterior wall (mm)**	0.6 ± 0.1	0.9 ± 0.1	0.9 ± 0.1	0.7 ± 0.1 **	0.8 ± 0.1 *
**End-systolic anterior wall (mm)**	0.9 ± 0.1	1 ± 0.1	1 ± 0.1	1 ± 0.2	1 ± 0.2
**End-diastolic posterior wall (mm)**	0.6 ± 0.1	0.8 ± 0.1	0.7 ± 0.1	0.7 ± 0.1	0.7 ± 0.1 *
**End-systolic posterior wall (mm)**	0.8 ± 0.1	0.9 ± 0.1	0.9 ± 0.1	1 ± 0.1	0.9 ± 0.1
**End-diastolic IV septum (mm)**	0.6 ± 0.1	0.9 ± 0.1	0.9 ± 0.2	0.8 ± 0.1 **	0.7 ± 0.1 **
**End-systolic IV septum (mm)**	0.8 ± 0.1	1 ± 0.2	1 ± 0.1	1 ± 0.2	1 ± 0.1
**End-diastolic LV internal diameter (mm)**	4.2 ± 0.2	5.2 ± 0.7	5 ± 0.6	4.5 ± 0.6 *	4.8 ± 0.7
**End-systolic LV internal diameter (mm)**	2.9 ± 0.2	4.4 ± 0.8	4.1 ± 0.7	3.3 ± 0.5 **	3.8 ± 0.8
**Heart rate (bpm)**	452.2 ± 59.8	479.2 ± 83.9	450 ± 93.5	479.5 ± 76.5	498.6 ± 82.4

Data are mean ± SD

*$P < 0.05$, **$P < 0.01$, ***$P < 0.001$ between *H19* overexpression with the respective AAV vector variant (AAV9 or AAV2-THGTPAD or AAV2- NLPGSGD) and TAC AAV9-empty; one-way ANOVA with Dunnett's multiple comparison to TAC AAV9-empty; IV – interventricular, LV – left ventricle
